# Disruption of Amino Acid Homeostasis by Novel ASCT2 Inhibitors Involves Multiple Targets

**DOI:** 10.3389/fphar.2018.00785

**Published:** 2018-07-19

**Authors:** Angelika Bröer, Stephen Fairweather, Stefan Bröer

**Affiliations:** Research School of Biology, Australian National University, Canberra, ACT, Australia

**Keywords:** SLC1A5, SLC38A1, SLC38A2, SLC7A5, amino acid transport, homeostasis, glutaminolysis

## Abstract

The glutamine transporter ASCT2 (SLC1A5) is actively investigated as an oncological target, but the field lacks efficient ASCT2 inhibitors. A new group of ASCT2 inhibitors, 2-amino-4-bis(aryloxybenzyl)aminobutanoic acids (AABA), were developed recently and shown to suppress tumor growth in preclinical *in vivo* models. To test its specificity, we deleted ASCT2 in two human cancer cell lines. Surprisingly, growth of parental and ASCT2-knockout cells was equally sensitive to AABA compounds. AABA compounds inhibited glutamine transport in cells lacking ASCT2, but not in parental cells. Deletion of ASCT2 and amino acid (AA) depletion induced expression of SNAT2 (SLC38A2), the activity of which was inhibited by AABA compounds. They also potently inhibited isoleucine uptake via LAT1 (SLC7A5), a transporter that is upregulated in cancer cells together with ASCT2. Inhibition of SNAT2 and LAT1 was confirmed by recombinant expression in *Xenopus laevis* oocytes. The reported reduction of tumor growth in pre-clinical models may be explained by a significant disruption of AA homeostasis.

## Introduction

Rapid growth of cancer cells requires the maintenance of a homeostatic pool of cytosolic AAs for protein biosynthesis and metabolic demands (Broer and Broer, 2017). Protein biosynthesis is an essential function for cancer cell growth, using all 20 proteinogenic AAs, and is limiting the overall growth of the tumor (Hosios et al., 2016). Membrane transporters are crucial to facilitate the entry of AAs into cells and also for AA signaling. Microarray data pointed to elevated expression levels of SLC1A5 (ASCT2) and SLC7A5 (LAT1) in many cancers (Fuchs and Bode, 2005), which since has been confirmed in many studies and cell lines (reviewed in Bhutia et al., 2015). To explain the abundance of ASCT2 and LAT1 in cancer cells, a model was proposed in which glutamine enters cells through ASCT2 and is subsequently used as an exchange substrate to import leucine among other essential AAs via LAT1 and to maintain mTORC1 in an activated state (Nicklin et al., 2009). The model has been challenged because ASCT2 deletion does not reduce mTORC1 signaling in several cell lines (Broer et al., 2016; Cormerais et al., 2018). As a result, we proposed an alternate model, in which ASCT2 and LAT1 play an important role in harmonizing AA pools, due to their rapid AA exchange activity (Broer et al., 2016; Broer and Broer, 2017). Regardless of the role, ASCT2 has been targeted by RNA-mediated silencing in a number of studies. Significant reduction of cell growth was observed in Sloan-Kettering hepatoma cells (Fuchs et al., 2004). Similarly, reduced growth and tumor progression was reported in PC-3 prostate cancer cells (Wang et al., 2015). van Geldermalsen et al. (2016) reported reduction of cell growth in HCC1806 basal-like breast cancer cells, but not in MCF-7 luminal cancer cells. ASCT2 knockdown also significantly reduced tumor size in HCC1806 xenografts. Hassanein et al. (2015) reported highly variable tumor size in A549 lung cancer cell xenografts, with very large tumors only occurring in cells containing ASCT2. When ASCT2 was deleted in A549 and LS174T colon cancer cells, *in vitro*, cell growth was reduced only in A549 cells, but xenograft tumor size of both cell lines was reduced (Cormerais et al., 2018).

**FIGURE 1:**
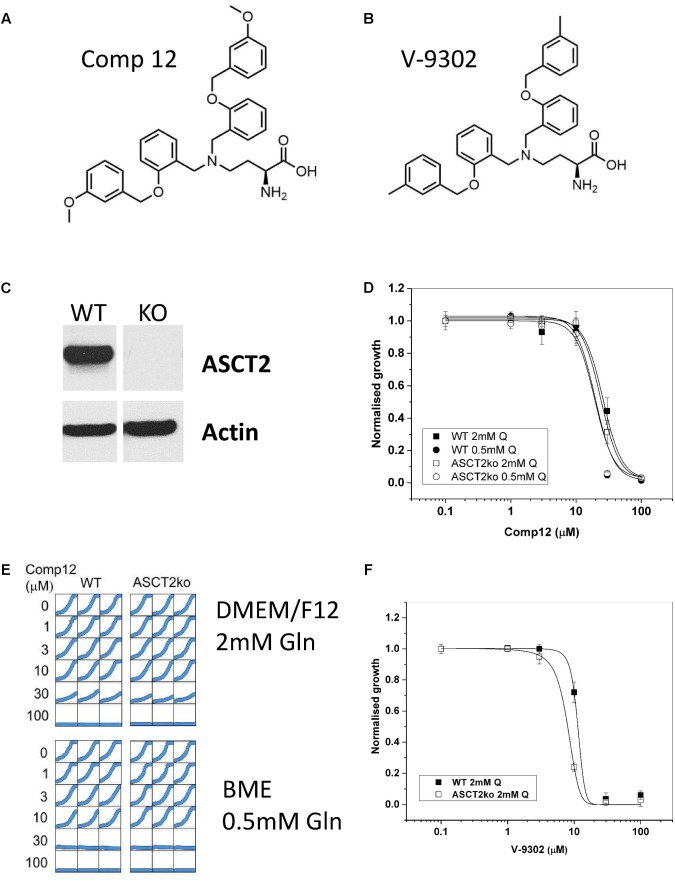
Inhibition of tumor cell growth by AABA. Structure of 2-amino-4-bis(aryloxybenzyl)aminobutanoic acids compound 12 **(A)** and V-9302 **(B)** as described by Schulte et al. (2016, 2018). **(C)** The presence of ASCT2 in wild-type and genome-edited 143B cells was evaluated by western blotting of cell homogenates using an ASCT2-specific antibody. **(D)** Growth of parental and ASCT2ko 143B cells was monitored using IncuCyte technology in the presence of increasing concentrations of compound 12 (*n* = 12, cells were seeded from at least three different batches). **(E)** Reproducibility of cell growth assays in a well-to-well comparison showing growth in DMEM/F12 supplemented with 2-mM glutamine and BME supplemented with 0.5-mM glutamine. Concentration of compound 12 is indicated in the margin. **(F)** Growth of parental and ASCT2ko 143B cells was monitored using IncuCyte technology in the presence of increasing concentrations of V-9302 (*n* = 10, cells were seeded from at least three different batches).

Pharmacological approaches to test the role of ASCT2 in cancer cell growth have been hampered by the lack of specific inhibitors. Several studies have used γ-glutamyl-*p*-nitroanilide (GPNA) to examine involvement of ASCT2 in cancer cell growth (e.g., Hassanein et al., 2013; Ren et al., 2015; Wang et al., 2015); however, the glutamine analog blocks a variety of glutamine transporters such as SNAT1, SNAT2 (Broer et al., 2016), and LAT1 (Chiu et al., 2017). Thus, it is unclear whether the reduction of growth observed in studies using GPNA is a result of ASCT2 inhibition. Due to the lack of specific inhibitors, it is often difficult to precisely align the activity of individual transporters with the uptake of radiolabeled substrates. However, inhibition by *N*-methyl-aminoisobutyric acid (MeAIB) in non-epithelial cells is a reliable indicator for the presence of SNAT1 and/or SNAT2, together known as system A activity (Broer, 2014). LAT1 activity can now be identified using the specific high-affinity inhibitor JPH203 (Wempe et al., 2012). A novel potentially specific inhibitor of ASCT2 was published recently (Schulte et al., 2016), which reduced tumor growth in mice *in vivo* (Schulte et al., 2018) and appeared to be a potent blocker of ASCT2. The series of compounds is based on AABA. In the earlier study (Schulte et al., 2016), a derivative called compound 12 was identified as the most potent inhibitor of ASCT2. In the *in vivo* experiments (Schulte et al., 2018), a slightly different compound from the same series was used, called V-9302. In this study, we used compound 12 and V-9302 (**Figures 1A,B**), which were reported to inhibit human ASCT2 with an IC_50_ of 7–10 μM (Schulte et al., 2016, 2018). Here, we report that compound 12 and V-9302 do not inhibit ASCT2, but rather block Sodium-neutral AA transporter 2 (SNAT2 and SLC38A2) and the large neutral AA transporter 1 (LAT1 and SLC7A5). This was observed in 143B osteosarcoma cells and HCC1806 breast cancer cells and confirmed by recombinant expression of SNAT1, SNAT2, ASCT2, and LAT1 in *Xenopus laevis* oocytes. The combined block of SNAT2 and LAT1 is likely to underlie the observed biological effects. A specific ASCT2 inhibitor remains to be identified.

## Materials and Methods

### Custom Synthesis of 2-Amino-4-Bis(aryloxybenzyl)aminobutanoic Acid Compound 12 and V-9302

Synthesis was performed as described by Schulte et al. (2016). The compounds were synthesized by Exclusive Chemistry, Obninsk, Russia. Compound identity was verified by LC-MS and ^1^H-NMR (Supplementary Figures S1, S2).

### Animals

Holding of *Xenopus laevis* frogs (purchased from Nasco, Fort Atkinson, WI, United States) and the surgical procedure to remove parts of the ovary were approved by the Animal experimentation ethics committee of the Australian National University (Protocol A2017/36). All procedures were carried out in accordance with the recommendations of the Australian code for the care and use of animals for scientific purposes.

### Cell Lines and Cell Culture

Human thymidine-kinase-negative osteosarcoma cells, 143B (TK–) were a gift by Dr. David Tscharke (John Curtin School of Medical Research, ANU), and human HCC1806 breast cancer cells were a gift by Dr. Jeff Holst (Centenary Institute, Sydney, NSW, Australia). Both cell lines were either cultured in DMEM/Ham’s F12 (Sigma 6124 supplemented with 2-mM glutamine) or in BME medium (Thermo 21010) supplemented with 10% dialyzed fetal bovine serum (FBS, Life Technologies), non-essential AAs (**Table 1**), and 0.5-mM sodium pyruvate at 37°C in a humidified atmosphere of 5% CO_2_ in air. For sub-culturing, cells were detached by trypsinization (0.05 or 0.25% trypsin–EDTA, GIBCO). Cell counting was performed using a Scepter cell counter (Millipore, United States) or a hemocytometer. All complete cell culture media were supplemented with 2-mM L-glutamine (GIBCO). Cell viability after trypsinization was generally ≥95% as evaluated by trypan-blue exclusion.

**Table 1:** Amino acid composition of media used in this study (in mM).

Amino acid	DMEM/F12 (Sigma D6421)	BME (Thermo 21010)	Physiological concentrations^#^
Arg	0.702	0.099	0.032–0.11
Cys	0.1	0.05	0.003–0.095
His	0.15	0.051	0.039–0.123
Ile	0.416	0.198	0.036–0.107
Leu	0.45	0.198	0.068–0.183
Lys	0.5	0.199	0.103–0.255
Met	0.234	0.05	0.004–0.044
Phe	0.214	0.1	0.035–0.08
Thr	0.445	0.2	0.085–0.231
Trp	0.044	0.0196	0.029–0.077
Tyr	0.23	0.099	0.031–0.09
Val	0.452	0.2	0.136–0.309
Asp	0.05	0.005*	<0.007
Gly	0.25	0.35*	0.126–0.49
Pro	0.15	0.25*	0.097–0.368
Ser	0.25	0.1*	0.063–0.187
Ala	0.05	0.5*	0.2–0.579
Asn	0.057	0.1*	0.037–0.092
Glu	0.05	0.1*	0.013–0.113
Gln	variable	variable	0.371–0.957

*^∗^AAs added to the media at the final concentration indicated in the table. ^#^Physiological plasma concentration adult, fasting (Mayo Clinic, Quantitative AA-Analysis). Glutamine was added to the media at the concentration indicated.*

### Genomic Mutation of the ASCT2 (Slc1a5) Gene

A commercial CRISPR/Cas9 system was used (Sigma). Generation of ASCT2ko 143B cells was described recently (Broer et al., 2016), and the same method was used to mutate ASCT2 in HCC1806 cells. Briefly, the construct U6gRNA-pCMV-Cas9-2A-GFP contains a 22-bp guide RNA (cctcgaagcagtcaacctcccg) resulting in cleavage/repair of the *Slc1a5* gene in exon 7. An endotoxin-free preparation (Macherey and Nagel) of the plasmid was used for transfection of HCC1806 cells maintained in DMEM/Ham’s F12/10% FBS/2-mM glutamine. Cells were seeded out in a 60-mm dish and grown until reaching 80% confluence. Immediately before transfection, the cells were replenished with fresh DMEM/HamF12/10% FCS/2 mM glutamine. Plasmid DNA (4 μg) and 10-μl Lipofectamine 2000 (Invitrogen) were separately incubated in 500 μl of Opti-MEM (Invitrogen) for 5 min at room temperature, before combining them and incubating for a further 20 min at room temperature to form complexes. The complexes were then added drop-wise to the cells and placed in an incubator at 37°C/5% CO_2_, followed by a media change after 6 h. After 48 h of expression, cells were trypsinized [0.25% Trypsin/EDTA (Invitrogen)] and collected by centrifugation (500 × *g*) followed by three washes in Dulbecco’s phosphate-buffered saline supplemented with 5-mM glucose and 1% dialyzed FBS (Sigma). Cells were then passed through a cell strainer (70 μm, Corning), centrifuged, and suspended in PBS (pH 7.4) supplemented with 5-mM glucose and 1% dialyzed FBS. Single cell sorting was performed using an ARIA II FACS machine (FACS Facility Australian National University) with GFP intensity set at two different levels. Single cells were collected in 96-well plates containing DMEM/Ham’s F12/10% FBS/2 mM glutamine and incubated for up to 3 weeks at 37°C/5% CO_2_. Established clones were trypsinized and seeded into 25-cm^2^ cell culture flasks (Corning) for further propagation. Inactivation of ASCT2 was verified by immunoblotting and genomic sequencing.

### Growth Assays

For growth assays, HCC1806 and 143B cells – derived from at least three different cell batches – were seeded into 96-well culture plates at a density of 4,000–5,000 cells per well and kept in DMEM/Ham’s F12/10% FCS or in BME/10% dialyzed FCS supplemented with 0.5 or 2-mM glutamine. Inhibitor (AABA) was added at the concentrations indicated in the figures. Cell proliferation was measured using an IncuCyte system (Essen BioScience) over 54 h with a scan interval of 3 h. IncuCyte data are shown as cell confluence (mean ± SEM) at set intervals.

### Amino Acid Flux Assays

All uptake assays were performed in a water bath at 37°C as described by Deitmer et al. (2003) with some modifications. Briefly, cells were grown to confluence in 35-mm cell culture dishes and the media was replaced 12 h before the uptake assay. For Na^+^-dependent transport, Hanks’ balanced salt solution (136.6 mM NaCl, 5.4 mM KCl, 2.7 mM Na_2_HPO_4_, 1.3 mM CaCl_2_, 0.5 mM MgCl_2_, 0.44 mM KH_2_PO_4_, 0.41 mM MgSO_4_, 5 mM HEPES, pH 7.5) supplemented with 5-mM glucose was used. For Na^+^-independent transport, NaCl was substituted with *N*-methyl-D-glucamine chloride (NMDG-Cl), and sodium phosphate with potassium phosphate. L-[U ^14^C]glutamine (1.85 MBq/mL, 9.26 GBq/mmol, PerkinElmer) and L-[U ^14^C]isoleucine (1.85 MBq/mL, 9.26 GBq/mmol, PerkinElmer) were used at ∼2,000 dpm/nmol with unlabeled L-AA adjusted to 100 μM final concentration. Transport rates were normalized to cell-derived protein content measured by Bradford reagent (Sigma). Uptake rates were expressed as mean (±SD) and statistical differences were evaluated by ANOVA or *T*-test.

### Preparation of Cell Homogenates and Surface Biotinylation

Cells were grown to approximately 80% confluency on a 100-mm cell culture dish in DMEM/F12/10% FCS 2-mM glutamine before washing the monolayer gently thrice with ice-cold PBS (pH 7.4). Cells were kept on ice at all times. To lyse the cells, 500 μl of RIPA Lysis Buffer (Sigma) supplemented with protease inhibitor Pefabloc EDTA-free (Roche) was added, and the cells collected with a cell scraper. The lysate was transferred to a reaction tube and incubated on ice for 15 min, before homogenization with an Omni Pro200 for 20 s. The samples were incubated on ice for an additional 15 min before centrifugation at 13,000 × *g* for 5 min at 4°C. The supernatant was collected and subjected to a protein determination assay (Bradford Reagent, Sigma). Fifty micrograms of the homogenate was loaded into each well of a polyacrylamide gel (Invitrogen). After separation by PAGE, proteins were transferred onto nitrocellulose membranes for immunodetection.

For surface biotinylation, cells were grown in 60-mm dishes and washed thrice in 5 ml modified PBS (supplemented with 1-mM CaCl_2_ and 0.6-mM MgCl_2_, pH 8.0). Cells were then covered with 2-ml 0.5 mg/ml EZ-link Sulfo-NHS-lc-Biotin (Thermo Fisher Scientific) in modified PBS (pH 8.0) and incubated for 30 min at room temperature on a rotary shaker at low speed. Biotinylation was terminated by washing thrice in modified PBS supplemented with 100-mM glycine, pH 8.0. Cells were scraped together, transferred to a 1.5-ml reaction tube and lysed by addition of 1 ml 150 mM NaCl, 1% Triton X-100, 20 mM Tris–HCl, pH 7.5. The homogenate was incubated on ice for 1.5 h to complete lysis. Subsequently, the lysate was centrifuged at 13,000 × *g* in a table-top centrifuge for 10 min and the supernate was transferred to a new tube. After protein determination, equal amounts of cell lysate were added to 150-μl high-capacity streptavidin agarose beads (Thermo). The beads were incubated overnight at 4°C on a rotary shaker before washing four times in lysis buffer. The streptavidin-agarose slurry was mixed with protein sample buffer and sample reducing reagent. After boiling for 5 min, 40-μl samples were loaded onto a polyacrylamide gel. After separation, proteins were transferred onto nitrocellulose membranes for immunodetection.

### SDS-PAGE and Western Blotting

To prepare protein samples for SDS-PAGE, 50–100 μg total protein was mixed with 4× LDS sample buffer (Invitrogen), 10× reducing agent (Invitrogen), and made up to a final volume of 20 μl using MilliQ water. Homogenate samples were then incubated at 70°C for 10 min before loading onto the gel. Electrophoresis was performed using 4–12% Bis–Tris polyacrylamide NuPAGE^®^ gels (Invitrogen), electrophoresed in an XCell *SureLock*^®^ Mini-Cell (Invitrogen) under reducing conditions according to standard manufacturer procedures. The SeeBlue Plus 2 pre-stained protein ladder (Invitrogen) was used to estimate the apparent molecular weight of proteins. Following SDS-PAGE, proteins were transferred onto nitrocellulose membranes (GE Healthcare) using the Mini Trans-Blot Electrophoretic Transfer Cell (Bio-Rad) according to the standard protocols. Blots were blocked for 2 h at room temperature (or overnight at 4°C) in 50 ml 10% (w/v) skim milk in PBS (pH 7.4) with 0.15% TWEEN 20 (PBS-T). After washing three times in PBS-T for 10 min each, the blots were incubated with the primary antibody for 2 h or overnight in 5-ml skim milk (2%, w/v) in PBS-T at dilutions listed in **Table 2**. Excess primary antibody was removed by washing three times with PBS-T. Blots were incubated with 5 ml of diluted secondary antibody for 2 h. After washing three times in PBS-T and a final rinse in PBS, reactive bands were detected by enhanced chemiluminescence, using Luminata Crescendo or Forte western HRP Substrate (Millipore Merck). For re-probing, the same blots were incubated for 30 min at 70°C in 50-ml stripping buffer (62.5 mM Tris–HCl (pH 6.8), 2% SDS, and 100 mM 2-mercaptoethanol). Membranes were then washed three times with PBS-T and blocked for 3 h using 10% (w/v) skim milk in PBS-T before re-probing with next antibody as described above.

**Table 2:** Source of antibodies and dilution for western blotting.

Target	Manufacturer	Dilution
ASCT2 (SLC1A5)	Cell Signaling Technology	1:3000, clone D7C12
SNAT1 (SLC38A1)	Millipore	1:2000
SNAT2 (SLC38A2)	Abcam	1:2000
Na^+^/K^+^-ATPase	Abcam	1:7500
Actin	Cell Signaling Technology	1:5000
Rabbit IgG (HRP conj)	GE Healthcare	1:1000–1:10,000
Mouse IgG (HRP conj)	Cell Signaling Technology	1:3000–1:5000

### Docking Studies

A homology model of human LAT1 was built based on the apo outside open conformation (5J4I) of the *E. coli* arginine–agmatine antiporter (Ilgu et al., 2016) an established prokaryotic model of LAT1 (Palacin et al., 2016). The profile-based alignment was generated using HHpred through the MPI bioinformatics toolkit (Zimmermann et al., 2017). The alignment was then used to generate a homology model through the Swiss Model server (Waterhouse et al., 2018). Docking of V-9302 was performed using the PyRX docking software (Dallakyan and Olson, 2015).

### Oocyte Expression Systems and Flux Experiments

*Xenopus laevis* oocytes were isolated and maintained as described previously (Broer, 2003). Selected oocytes were injected with 10 ng of human ASCT2, human SNAT1, and human SNAT2 or 5 ng each of rat LAT1 and human 4F2hc cRNA and were used as described previously (Broer et al., 1999).

## Results

### Effect of AABA on Cell Growth

The structure of the two AABA derivatives evaluated in this study, compound 12 and V-9302, are depicted in **Figures 1A,B**, respectively. To study the role of ASCT2 in AA homeostasis, we have recently generated a 143B osteosarcoma cell line lacking ASCT2 using CrispR/Cas9-mediated genome editing (Broer et al., 2016; **Figure 1C**). The cell line was chosen to study the role of AA transporters in AA homeostasis due to its relatively simple expression profile of AA transporters. In addition, it can be used to test the specificity of ASCT2 inhibitors. To test the specificity of compound 12, we determined the growth rate of ASCT2 expressing parental cells and ASCT2ko cells (**Figures 1D,E**). To our surprise, growth was inhibited by compound 12 in both cell lines with equal efficacy (IC_50_ = 26 and 24 μM for parental and ASCT2ko, respectively, difference not significant), suggesting that the target of the compound was not ASCT2. This was confirmed using the related compound V-9302, which if anything inhibited ASCT2ko cells more effectively than the ASCT2 expressing parental cells (**Figure 1F**). To exclude that the inhibitor may not be effective at the high AA concentrations of DMEM/F12 medium (containing 2-mM glutamine), we repeated the experiment with compound 12 in BME medium with 0.5-mM glutamine, which is close to *in vivo* conditions (**Figures 1D,E**). The reduced competition by medium AAs improved the efficacy of the inhibitor slightly, but again no difference was observed between ASCT2 expressing and ASCT2ko cells (IC_50_ = 20 and 19 μM for parental and ASCT2ko, respectively, difference not significant).

### Effect of Compound 12 on Glutamine Transport

**FIGURE 2:**
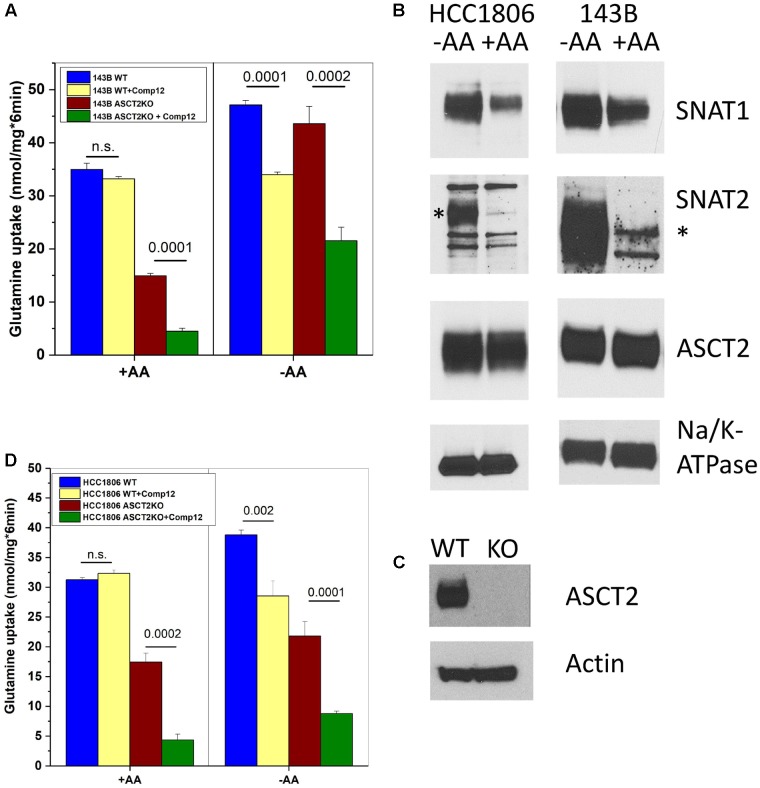
Inhibition of glutamine transport by AABA in wild-type and ASCT2ko cells. **(A)** Uptake of [^14^C]glutamine (100 μM) was measured in 143B cells [parental (wt) and ASCT2ko] in the presence or absence of 100 μM compound 12. Before transport, cells were kept in DMEM/F12 medium supplemented with 10% FCS (+AA) or in Hank’s buffered salts solution supplemented with 5-mM glucose and 1% dialyzed FCS (–AA) for 12 h [parental (wt) *n* = 5 and ASCT2ko *n* = 9 for each condition]. *p*-Values are indicated in the figure. **(B)** 143B cells and HCC1806 cells were incubated as in **A** and used for surface biotinylation and subsequent detection of glutamine transporters by immunoblotting. Na^+^, K^+^-ATPase was used as a loading control; the SNAT2-specific band is indicated by an asterisk (*) (*n* = 3). **(C)** Detection of ASCT2 in cell homogenates of parental and genome edited HCC1806 triple-negative breast cancer cells. Actin was used as a loading control (*n* = 5). **(D)** Uptake of [^14^C]glutamine (100 μM) was measured in HCC1806 cells [parental (wt) and ASCT2ko] in the presence or absence of 100 μM compound 12. Before transport, cells were kept in DMEM/F12 medium supplemented with 10% FCS (+AA) or in Hank’s buffered salts solution supplemented with 5-mM glucose and 1% dialyzed FCS (–AA) for 12 h (parental *n* = 5 and ASCT2ko *n* = 5 for each condition). *p*-Values are indicated in the figure.

ASCT2 is highly expressed in almost any cancer cell line and mediates a large fraction of glutamine uptake (Fuchs and Bode, 2005; Broer and Broer, 2017; Cormerais et al., 2018). To investigate the target of compound 12 further, we determined its effect on glutamine transport at a concentration of 100 μM, which is 5× the IC_50_ to inhibit growth (**Figure 1D**). In 143B cells, ASCT2 mediates about 50% of glutamine uptake at a concentration of 100 μM (Broer et al., 2016; also compare parental and ASCT2ko glutamine transport in **Figure 2A**). However, only a small non-significant fraction of glutamine uptake was inhibited by compound 12 in ASCT2 expressing parental 143B cells (**Figure 2A**, +AA panel, blue and yellow bars). In ASCT2ko cells, by contrast, the remaining glutamine uptake was strongly inhibited by compound 12 (**Figure 2A**, +AA panel, brown and green bars). We have recently shown that deletion of ASCT2 generates an AA stress response in 143B cells, which results in the upregulation of SNAT1 (SLC38A1) and SNAT2 (SLC38A2), collectively known as system A activity (Broer et al., 2016). Because SNAT1, SNAT2, and ASCT2 have similar substrate specificity, upregulation of these transporters can compensate for the lack of ASCT2 and it is often difficult to discriminate the individual transporters. To further investigate whether compound 12 inhibits SNAT1 and SNAT2 activity, we incubated cells overnight in Hank’s buffered salts solution [pH, 7.5 supplemented with 5-mM glucose and 1% dialyzed FCS, no AAs (–AA in **Figure 2**)], a maneuver that increases system A activity. We confirmed by surface biotinylation and western blotting that this treatment strongly increased the surface expression of SNAT1 and SNAT2, while ASCT2 remained unaffected (**Figure 2B**). The corresponding additional transport activity was completely abolished by addition of compound 12 in parental cells and in ASCT2ko cells (**Figure 2A**, +AA panel). Notably, induction of system A activity was more pronounced in ASCT2ko cells than in parental cells.

To substantiate our observation, we deleted ASCT2 in HCC1806 triple-negative breast cancer cells, a cell line also used by Schulte et al. (2018; **Figure 2C**). In contrast to 143B cells which are not sensitive to ASCT2 deletion (Broer et al., 2016), HCC1806 cells were reported to be sensitive to ASCT2 silencing (van Geldermalsen et al., 2016). Nevertheless, the ASCT2ko cells could be grown in DMEM/F12 medium. Very similar to 143B cells, glutamine uptake in ASCT2 expressing HCC1806 cells was not sensitive to inhibition by compound 12, but the remaining glutamine uptake in ASCT2ko cells was sensitive to the inhibitor (**Figure 2D**, panel +AA). AA depletion (**Figure 2D**, −AA) induced additional glutamine uptake capacity in parental cells, which was also sensitive to inhibition by compound 12. In ASCT2ko cells, glutamine transport did not increase much further upon AA depletion, but remained sensitive to compound 12 (**Figure 2D**). These results suggest that compound 12 does not target ASCT2 as suggested previously (Schulte et al., 2016), but may inhibit system A activity.

### Inhibition of System A and L by AABA

**FIGURE 3:**
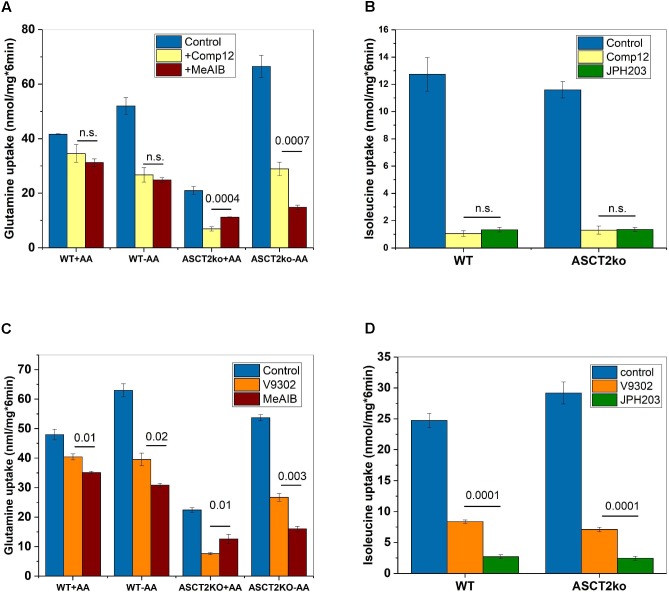
Inhibition of system A and LAT1 activity by AABA. **(A)** Uptake of [^14^C]glutamine (100 μM) was measured in parental (wt) or ASCT2ko 143B cells in the presence (*n* = 9 each group) or absence of 100 μM compound 12 (*n* = 9 each group) or 10-mM MeAIB (*n* = 6). Before transport, cells were kept in DMEM/F12 medium supplemented with 10% FCS (+AA) or in Hank’s buffered salts solution plus 5-mM glucose and 1% dialyzed FCS (–AA) for 12 h. n.s. difference not significant. *p*-Values for significant differences are shown in the figure. **(B)** Uptake of [^14^C]isoleucine (100 μM) was measured in Na^+^-free buffer in 143B cells (*n* = 6), in the presence (*n* = 6) or absence (*n* = 6) of 100 μM compound 12 or 2 μM JPH203 (*n* = 6) n.s. difference not significant. **(C)** Uptake of [^14^C]glutamine was measured as in **A**, but V-9302 was used instead of compound 12 (*n* = 3, *p*-values indicated in the figure). **(D)** Uptake of [^14^C]isoleucine was measured as in **B**, but V-9302 was used instead of compound 12 (*n* = 3, *p*-values indicated in the figure).

To investigate whether compound 12 inhibited system A activity, we compared it to MeAIB a known low-affinity inhibitor of system A activity, which is mediated by SNAT1 and SNAT2 (Christensen et al., 1965; Broer, 2014). Consistent with compound 12 being a system A inhibitor, the fraction of glutamine transport inhibited by MeAIB closely matched the fraction inhibited by compound 12, particularly in parental cells (**Figure 3A**, compare yellow and brown bars). Inhibition of glutamine transport was also similar after AA depletion, thereby resulting in an increased fraction of glutamine transport mediated by SNAT1/2. Small differences between MeAIB and compound 12 were observed in ASCT2ko cells, pointing to the upregulation of additional glutamine transporters (**Figure 3A**). Schulte et al. (2018) reported that leucine transport was inhibited by V-9302, although with lower potency than glutamine transport. We could reproduce this observation by showing potent inhibition of Na^+^-independent transport of isoleucine by compound 12 (**Figure 3B**). The inhibition was overlapping with the inhibition of isoleucine transport by the LAT1 specific inhibitor JPH203 (Wempe et al., 2012), demonstrating LAT1 as a second target. To test whether V-9302 (**Figure 1B**), which was shown to inhibit tumor growth *in vivo* (Schulte et al., 2018), has the same targets, we repeated the experiments. Consistent with the results shown in **Figure 3A** for compound 12, V-9302 inhibited only a small fraction of glutamine uptake in wild-type cells that was similar to the fraction inhibited by MeAIB (**Figure 3C**, compare orange and brown bars). AA depletion (-AA) increased the fraction of MeAIB and V-9302 sensitive glutamine uptake consistent with induction of SNAT1 and 2. In ASCT2ko cells, V-9302 and MeAIB inhibited a larger fraction of glutamine uptake than in parental cells, due to induction of SNAT1 and 2. Some differences between V-9302 and MeAIB can be observed in **Figure 3C**, which may be the result of changes in LAT1 activity. As reported, V-9302 inhibited isoleucine transport via LAT1, although it was performing less well than JPH203 (**Figure 3D**).

**FIGURE 4:**
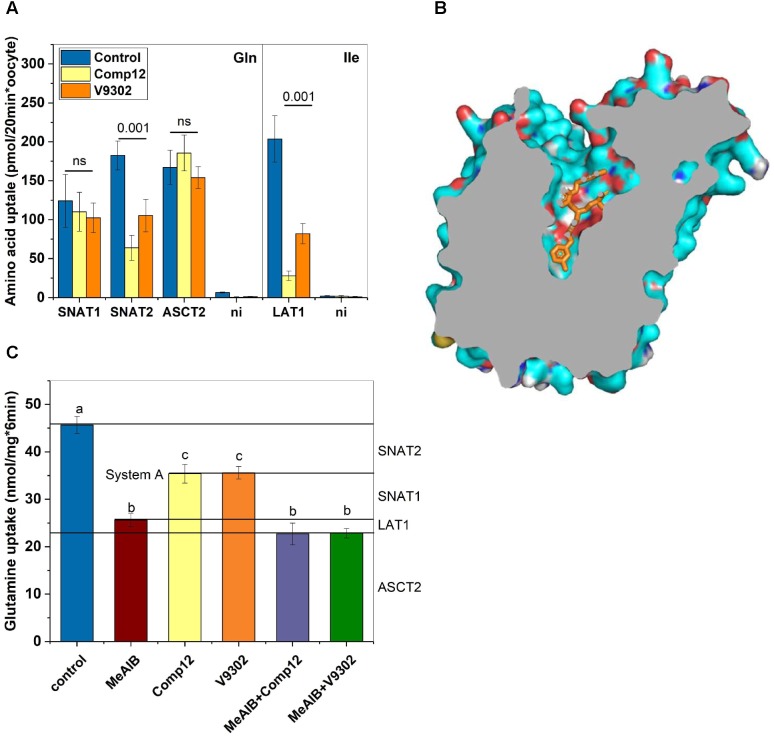
Evaluation of inhibitor specificity. **(A)** Transporters hSNAT1, hSNAT2, hASCT2, and h4F2hc/rLAT1 were expressed in *Xenopus laevis* oocytes. Transport experiments were performed 4–7 days after injection of the corresponding cRNAs. Glutamine uptake was used to measure hSNAT1, hSNAT2, and hASCT2 activity, while isoleucine transport was used to measure h4F2hc/rLAT1 activity. Uptake was measured in the presence or absence of inhibitors as indicated (*n* = 8 for each transporter) in three batches of oocytes. **(B)** A homology model of human LAT1 was generated after alignment with the *E. coli* arginine–agmatine antiporter (PDB 5J4I). Docking of V-9302 was performed using PyRX. One of several energetically favorable poses is shown. **(C)** Delineation of glutamine transport pathways in 143B osteosarcoma cells. The contributions of SNAT1, SNAT2, ASCT2, and LAT1 were deduced using combinations of inhibitors (*n* = 6). Groups that were different (*p* < 0.001) have different letter labeling.

The experiments thus far established that AABA compounds are potent inhibitors of several AA transporters in two different cell lines. To evaluate the effect on individual transporters we used recombinant expression in *Xenopus laevis* oocytes, a system widely used to express membrane transporters (**Figure 4A**). Consistent with our cell line studies, we found that compound 12 and V-9302 inhibited SNAT2 and LAT1, but not ASCT2 or SNAT1 (**Figure 4A**). In order to understand the potent inhibition of LAT1, we performed molecular docking studies on a homology model of LAT1 based on an alignment with the arginine–agmatine antiporter from *E. coli*. This transporter is an established model for LAT1 (Palacin et al., 2016) and its structure is available in the outside-open conformation (Protein data bank structure 5J4I; Ilgu et al., 2016), which is the relevant conformation for inhibitor binding (Broer, 2018). Several binding poses with similar binding energies were identified, demonstrating a tight fit of the inhibitor to the vestibule and substrate binding site of the outside open transporter (**Figure 4B**).

In most cancer cells, glutamine uptake is mediated by contributions from ASCT2, SNAT1, SNAT2, and LAT1 (Broer and Broer, 2017). A comprehensive understanding of the inhibitory properties of compound 12 and V-9302 should allow a seamless delineation of these pathways. This is shown in **Figure 4C** using 143B wild-type cells that were incubated in AA-free media to induce SNAT2. MeAIB was used to inhibit SNAT1 and SNAT2 together (known as system A activity). Compound 12 and V9302 inhibited about 50% of system A activity, attributing the other 50% to SNAT1. Combining MeAIB with V9302 or compound 12, showed a slightly stronger depression of glutamine uptake than MeAIB alone, an activity which can be attributed to LAT1. LAT1 has a low affinity for glutamine (Napolitano et al., 2015) and therefore its contribution is rather small consistent with our previous data (Broer et al., 2016). The fraction of glutamine uptake mediated by ASCT2 remained resistant to any of the inhibitors.

## Discussion

**FIGURE 5:**
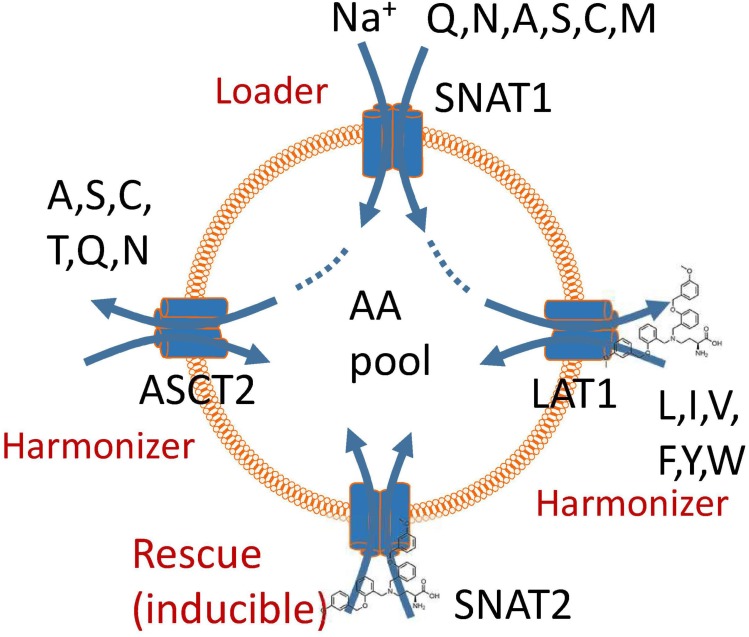
A model of amino acid homeostasis in cancer cells. The physiological role of SNAT1 is to accumulate non-essential amino acids in the cytosol (loader). These are used as exchange substrates to bring the remaining neutral amino acids into the cytosol via exchangers ASCT2 and LAT1, thus harmonizing amino acid pools. Blockade of LAT1 causes a rescue response resulting in the induction of SNAT2. Block of these two transporters impairs accumulation and harmonization of amino acid pools in cancer cells and also negates the rescue response.

Despite the urgent need for a high-affinity, specific ASCT2 inhibitor to study the role of AA transporters in cancer cell growth, our results demonstrate that neither compound 12 nor V-9302 are fulfilling this role. The strong effect of V-9302 on tumor cell growth is consistent with the combined inhibition of SNAT2 and LAT1, but may involve additional targets. We have recently proposed a unified model of AA homeostasis (Broer et al., 2016; **Figure 5**) in which SNAT1 serves as an AA “loader” that accumulates a small group of non-essential neutral AAs into cancer cells. These serve as exchange substrates for AA antiporters such as ASCT2 and LAT1 (Utsunomiya-Tate et al., 1996; Meier et al., 2002; Pingitore et al., 2013; Napolitano et al., 2015), which bring essential AAs [threonine (via ASCT2), and leucine, isoleucine, valine, phenylalanine, tyrosine, tryptophan, and histidine (via LAT1)] into the cells at the expense of non-essential AAs, particularly glutamine. We termed these antiporters “harmonizers” to emphasize their role in AA homeostasis and the maintenance of significant pools of all 20 proteinogenic AAs. Under conditions of nutrient limitation, cancer cells exert an AA stress response, which results in the upregulation of SNAT2 as shown in this study and previously (Lopez-Fontanals et al., 2003; Palii et al., 2006). Moreover, it results in the induction of anabolic pathways for non-essential AAs (Balasubramanian et al., 2013). The block of LAT1 alone by V-9302 is sufficient to disrupt AA homeostasis, due its role in the acquisition of essential AAs. The rescue response, however, is blunted by the concurrent inhibition of SNAT2. The efficacy of V-9302 is likely to be a result of inhibition of the harmonizer LAT1 in combination with the “rescue” transporter SNAT2. However, we cannot exclude additional targets of this compound. Overall, our results explain that potent disruption of AA homeostasis in cancer cells can be used to block tumor cell growth.

In two cancer cell lines (143B and HCC1806), we have shown that ASCT2 can be deleted without compromising cell growth *in vitro* (data presented here and in Broer et al., 2016). This was also confirmed in another study using LS174T colon cancer and A549 lung cancer cells (Cormerais et al., 2018). These results suggest that therapeutic approaches targeting only one particular AA transporter are unlikely to succeed. However, disturbing AA homeostasis more drastically remains a promising approach in cancer therapy. Essential AAs must be acquired by cancer cells through membrane transport. This is illustrated by the deletion of CD98 in LS174T cells, a surface protein required for the trafficking of a number of AA transporters, such as LAT1, LAT2, and xCT to the cell surface (Cormerais et al., 2016). Surprisingly, the small residual uptake activity mediated by LAT1 in the absence of CD98 (about 10%) appeared to be sufficient to sustain cell growth. Complete block by addition of the LAT1-specific inhibitor JPH203 or deletion of LAT1 did reduce cell growth dramatically. This suggests that cancer cell lines have significant reserve capacity for AA uptake, the uptake of which needs to be inhibited completely to block cell growth. This was confirmed in a first clinical trial using JPH203, in which only patients receiving higher doses of the drug achieved longer survival (Okano et al., 2018). The *in vivo* efficacy of V-9302 suggests that it causes sufficient disruption to AA homeostasis, potentially in combination with other effects, to be a promising candidate for cancer therapy, but its actions cannot be explained by inhibition of ASCT2.

## Author Contributions

AB and SF performed the experiments and prepared figures for publication. SB developed the concept of the study, designed the experiments, and wrote the manuscript.

## Conflict of Interest Statement

The authors declare that the research was conducted in the absence of any commercial or financial relationships that could be construed as a potential conflict of interest.

## Abbreviation

AA: amino acid
AABA: 2-amino-4-bis(aryloxybenzyl)aminobutanoic acids
DMEM: Dulbecco’s modified Eagle’s medium.
