# Supplementary material for: Disruption of Amino Acid Homeostasis by Novel ASCT2 Inhibitors Involves Multiple Targets
Source: Front Pharmacol. 2018 Jul 19;9:785. doi: 10.3389/fphar.2018.00785 (PMC6060247; doi:10.3389/fphar.2018.00785)

Figure S1: Compound characteristics

A) LC-MS of 2-Amino-4-bis(aryloxybenzyl)aminobutanoic acid *compound 12*

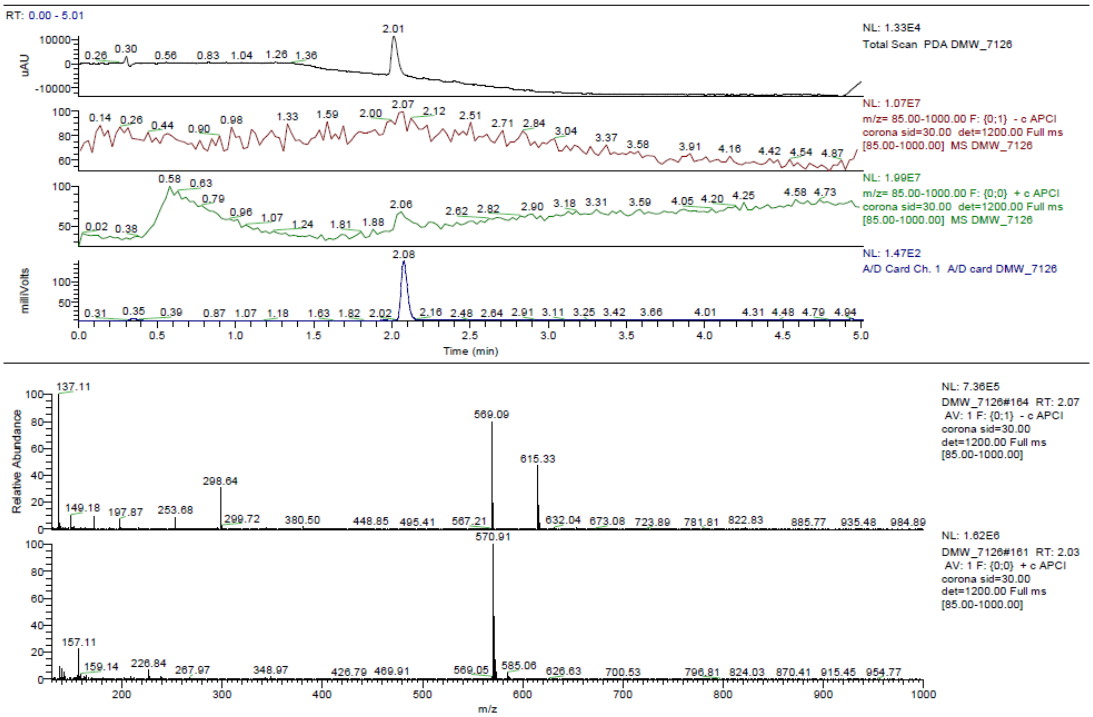

B) Proton NMR of 2-Amino-4-bis(aryloxybenzyl)aminobutanoic acid *compound 12* *(solvent $CDCl_3$)*

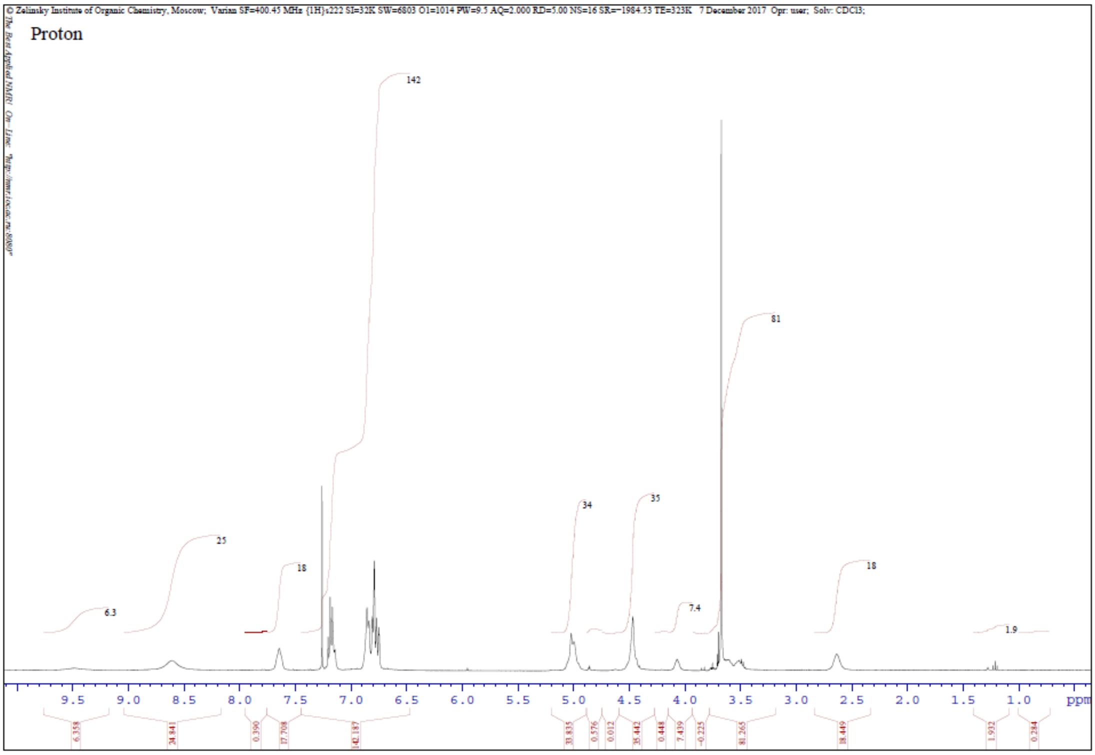

Figure S2: Compound characteristics

A) LC-MS of 2-Amino-4-bis(aryloxybenzyl)aminobutanoic acid *compound V-9302*

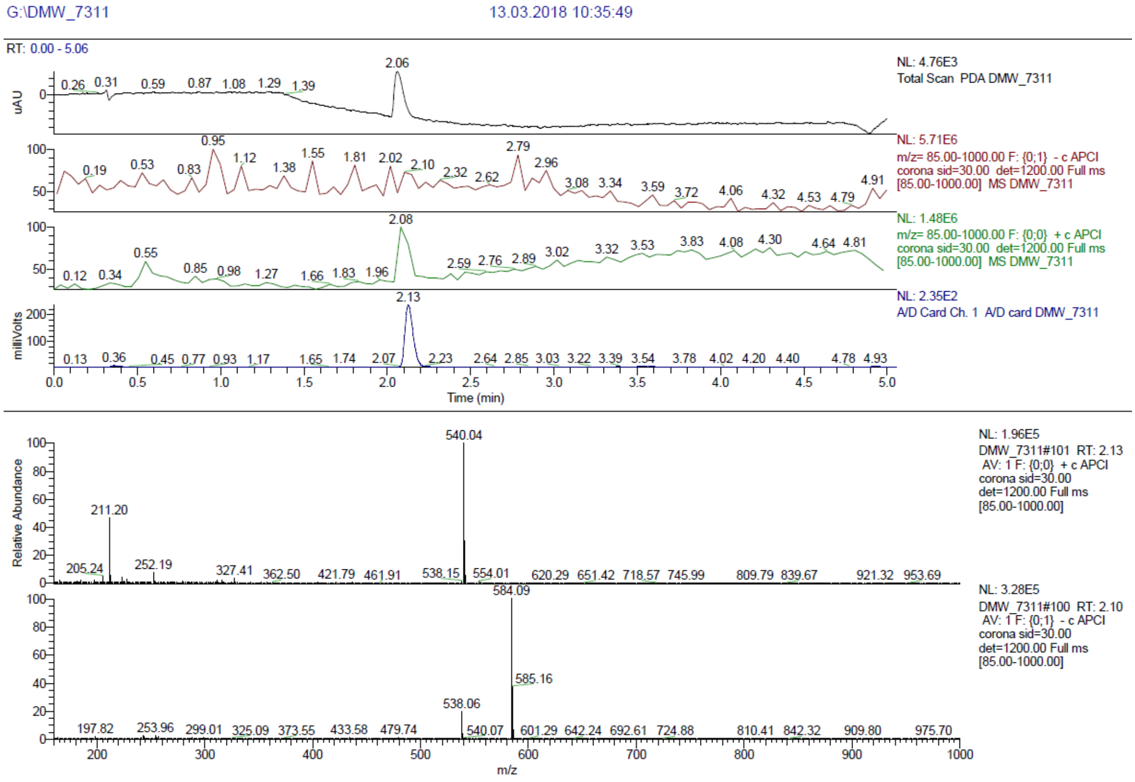

B) Proton NMR of 2-Amino-4-bis(aryloxybenzyl)aminobutanoic acid *compound V-9302 (solvent DMSO-D6)*

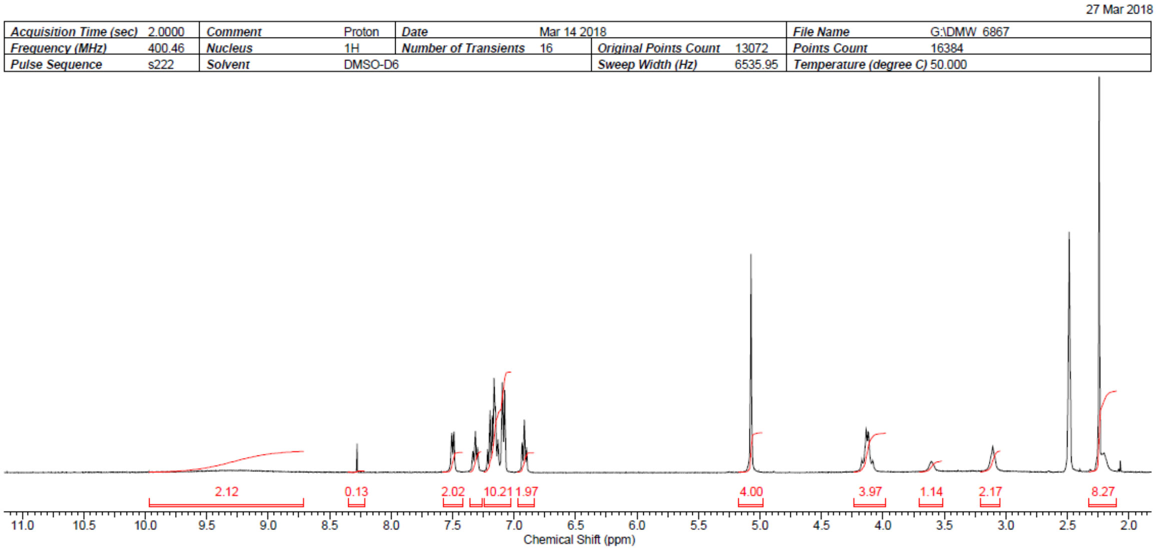

Supplement: Supplementary file 1 [file Data_Sheet_1.pdf]
